# Inhaled corticosteroid use is associated with increased circulating T regulatory cells in children with asthma

**DOI:** 10.1186/1476-7961-11-1

**Published:** 2013-01-25

**Authors:** Anne Marie Singh, Paul Dahlberg, Kristjan Burmeister, Michael D Evans, Ronald Gangnon, Kathy A Roberg, Christopher Tisler, Douglas DaSilva, Tressa Pappas, Lisa Salazar, Robert F Lemanske, James E Gern, Christine M Seroogy

**Affiliations:** 1 Departments of Pediatrics, University of Wisconsin, 1111 Highland Avenue, 4139 WIMR, Madison, WI 53705-2275, USA; 2 Departments of Biostatistics and Medical Informatics, University of Wisconsin, Madison, WI, USA; 3 Departments of Population Health Sciences, University of Wisconsin, Madison, WI, USA; 4 Departments of Medicine, University of Wisconsin, Madison, WI, USA; 5 Northwestern Feinberg School of Medicine, Chicago, IL, USA

**Keywords:** Asthma, CD127, Foxp3, Inhaled corticosteroids, T regulatory cell

## Abstract

**Background:**

T regulatory (Treg) cells are important in balancing immune responses and dysregulation of Treg cells has been implicated in the pathogenesis of multiple disease states including asthma. In this study, our primary aim was to determine Treg cell frequency in the peripheral blood of children with and without asthma. The secondary aim was to explore the association between Treg cell frequency with allergen sensitization, disease severity and medication use.

**Methods:**

Peripheral blood mononuclear cells from healthy control subjects (N = 93) and asthmatic children of varying disease severity (N = 66) were characterized by multi-parameter flow cytometry.

**Results:**

Our findings demonstrate that children with asthma had a significantly increased frequency of Treg cells compared to children without asthma. Using a multivariate model, increased Treg cell frequency in children with asthma was most directly associated with inhaled corticosteroid use, and not asthma severity, allergic sensitization, or atopic status of the asthma.

**Conclusion:**

We conclude that low dose, local airway administration of corticosteroids is sufficient to impact the frequency of Treg cells in the peripheral blood. These data highlight the importance of considering medication exposure when studying Treg cells and suggest inhaled corticosteroid use in asthmatics may improve disease control through increased Treg cell frequency.

## Introduction

The CD25+ T regulatory (Treg) cell, a widely accepted T cell subset with immune suppressive properties, was initially thought to exist primarily to prevent autoimmune diseases [1]. Increasing evidence suggests that Treg cells are important in varied immune responses, including allergic diseases [2-6]. A role for Treg cells in allergic disease was first highlighted in Immunodysregulation, Polyendocrinopathy, Enteropathy, X-linked (IPEX) patients [7,8]. IPEX patients lack Treg cells and have increased serum IgE levels, skewing of T cell responses toward a Th2 phenotype, and autoimmune endocrinopathies [9]. In allergic disease, the current concept suggests that inadequate Treg cell suppression of effector cells or diminished Treg cell numbers contributes to the development and perpetuation of allergic inflammation [10,11]. Studies utilizing murine models of allergic asthma provide strong evidence for an essential Treg cell role in modulating allergic airway inflammation [12-16]. Two recent clinical studies have suggested that Treg cells in adult asthmatic subjects are elevated while another study demonstrated diminished Treg cells in asthmatic children [17-19]. Therefore, the role of Treg cells in allergic asthma remains poorly defined, particularly in children when the initiation of allergic asthma most often occurs.

As Treg cells are important for suppressing immune responses and inflammation, we hypothesized that children with asthma would have fewer Treg cells compared to unaffected children. One of the challenges of identifying Treg cells is the lack of a unique marker. Published studies analyzing human Treg cells have utilized several phenotypic markers including CD25, CD127, and Foxp3. In this study, we used multi-parameter flow cytometry to investigate the frequency of Treg cells in the peripheral blood of children ages 7–8 years with or without asthma. The frequency of Treg cells was further analyzed in relationship to disease severity, allergic sensitization and medication use.

## Methods

### Study subjects and design

Analysis of peripheral blood mononuclear cells (PBMC) from 159 children ages 7–8 years was performed using flow cytometry. Subjects were enrolled in the Childhood Origins of ASThma (COAST [20]) study at birth (N = 141) or were newly recruited based on a diagnosis of asthma (N = 18). COAST is a high-risk birth cohort in that at least 1 parent was required to have a history of physician-diagnosed asthma and/or documented allergen sensitization (defined as one or more positive aeroallergen skin prick tests) [20,21]. Flow cytometry was performed in children enrolled in COAST over a one-year period. Children within the cohort were classified as having asthma as defined below. Additional children with a physician diagnosis of asthma were recruited to enrich the sample for children with asthma, and were age-matched to those enrolled in the COAST birth cohort. An effort was made to enroll equal numbers of children with intermittent, mild persistent and moderate/severe persistent asthma. This study was approved by University of Wisconsin Human Subjects Committee and legal guardians of all subjects provided informed consent.

### Asthma definition and severity

Asthma was diagnosed as previously described by study physicians using National Asthma Education and Prevention Program (NAEPP) Expert Panel Report Guidelines [21,22]**.** Medication use was assessed by questionnaire at the study visit. Since asthmatic children were taking medication at enrollment, asthma classification was based on medication use and symptom control. Classification was as follows: 1) children well-controlled without a daily controller medication were classified as intermittent; 2) children well-controlled on low dose ICS or leukotriene receptor antagonist were classified as mild persistent; 3) children requiring medium dose ICS or two classes of controller medication for asthma (ICS + long acting beta agonist, medium dose ICS or medium-high dose ICS + leukotriene receptor antagonist) were classified as moderate persistent; and 4) children requiring high dose ICS plus long-acting beta agonist were classified as severe persistent (medication use in each subject as it relates to asthma severity is listed in Additional file 1). Spirometry was performed using the Jaeger MasterScreen system at the study visit. Eigen criteria for lung function were used, similar to those used in published studies [23,24]. If the child was ill, requiring albuterol or with asthma symptoms, the visit was rescheduled. Respiratory viral surveillance nasal samples were collected on all subjects for another study. Virology data from a subset of the study patients (N = 49) was analyzed for respiratory viral infections and symptoms were scored as previously described [22]. Respiratory viral panel analysis from 49 subjects demonstrated no viral detection in 43 subjects and 6 subjects were virus positive (2 human rhinovirus, 2 coronavirus, 1 RSV, and 1 influenza). Additionally, symptom scores from 134 of the 159 study subjects were obtained. The majority of subjects (N = 110, 82%) denied recent respiratory infection symptoms at the time of blood draw for flow cytometry. 10 of those 134 were symptomatic: 7 mild (symptom score 1–4) and 3 moderate (symptom score 5–6). All 10 had respiratory viral panel analysis. Viral detection rates were 67% (2/3) for moderate illness, 29% (2/7) for mild illness, and 5% (2/39) for asymptomatic visits.

### Flow cytometry

PBMCs were isolated using Ficoll density gradient, incubated with Fc block (eBiosciences, San Diego, CA) and then stained in media with the following antibodies: CD3-Pacific Blue (clone UCHT1, eBiosciences), CD4-PerCP (clone RPA-T4), CD25-APC (clone 2A3), CD127-PE (clone hIL-7R-M21) (BD Biosciences, San Jose, CA) and FoxP3-Alexa Fluor 488, (clone 206D, BioLegend, San Diego, CA, USA) according to manufacturer’s protocol within 24 hours of blood draw. Cells were acquired on a LSR II (BD Biosciences). Positive staining and gating strategy was determined by comparison to isotype or Fluorescence Minus One (FMO) control. Data were analyzed using Flowjo software (Treestar, San Carlos, CA).

### Total IgE and allergy tests

Total and allergen-specific IgE for birch, grass mix, ragweed, *Dermatophagoides pteronyssinus, Dermatophagoides farinae, Alternaria alternata,* cat, dog, cockroach, egg, and peanut were analysed by UniCAP 100 fluoroenzyme immunoassay (FEIA, Pharmacia and Upjohn Diagnostics, Kalamazoo, MI). The sensitivity for detection of total IgE was 2 kU/L. Allergen-specific IgE values of ≥ 0.35 kU/L were considered positive. Birch, grass mix, ragweed were considered seasonal allergens and dust mite, cat, dog, alternaria, and cockroach were considered perennial allergens.

### Statistical analysis

Demographic characteristics were compared by asthma diagnosis using chi-square tests for association. The frequency of Treg cells was compared using one-factor (asthma, asthma with ICS, asthma severity, atopic asthma, FEIA) and two-factor (asthma severity & ICS, atopic asthma & ICS, FEIA & ICS) ANOVA models. Results of two-factor ANOVA models are summarized using least squares means (estimated marginal means for a balanced population). The Treg cell percentage is displayed in the figures as mean ± 95% confidence interval (CI) or standard deviation (SD). The association between Treg cell percentage and total IgE (log-transformed) was analyzed using Pearson’s correlation coefficient. A two-sided p-value < 0.05 was regarded as significant.

## Results

### Study population

Table 1 outlines the characteristics of the study subjects. The percentage of children with asthma was 42% with no significant difference in gender. The children with asthma were more likely to be African American. There were trends for inverse associations between FEV 0.5, PEF and FVC and asthma disease severity. Asthma diagnosis and disease severity were significantly associated with perennial allergen sensitization and total IgE, but not seasonal allergen sensitization (Table 1). Additionally, body mass index (BMI) was recorded for 66% of the subjects at the time of blood draw for Treg cell analysis. Within this subset, there is no difference in BMI by asthma status or ICS use (data not shown).

**Table 1:** Characteristics of study subjects

**Disease classification**	**No asthma**	**Intermittent**	**Mild persistent**	**Moderate/severe persistent**	**p value**
Number of subjects	93	31	15	20	
Age(years) (±SD)	7.6 ± 0.8	7.6 ± 0.5	7.4 ± 0.8	7.4 ± 1.0	0.54
				
Female	46 (49%)	10 (32%)	6 (40%)	8 (40%)	0.38
Male	47 (51%)	21 (68%)	9 (60%)	12 (60%)	
Caucasian	89 (96%)	29 (94%)	11 (73%)	16 (80)	0.008
African American	3 (3%)	3 (10%)	6 (40%)	6 (30%)	<0.0001
American Indian	4 (4%)	0 (0%)	0 (0%)	0 (0%)	0.84
Asian	0 (0%)	1 (3%)	0 (0%)	0 (0%)	0.42
Hispanic or Latino	4 (4 %)	1 (3%)	1 (7%)	0 (0%)	0.83
Spirometry(median [25^th^, 75^th^])					
FEV1	1.50 [1.37, 1.72]	1.54 [1.27, 1.66]	1.23 [1.09, 1.63]	1.46 [1.29, 1.83]	0.11
FEV1% pred	103 [95, 109]	103 [89, 113]	90 [85, 101]	102 [94, 107]	0.43
FVC	1.85 [1.64, 2.06]	1.81 [1.60, 2.05]	1.62 [1.31, 1.93]	1.88 [1.51, 2.21]	0.06
FVC% pred	108 [100, 115]	110 [98, 118]	96 [94, 116]	114 [105, 120]	0.19
FEV0.5	1.11 [1.01, 1.30]	1.14 [0.97, 1.29]	0.92 [0.78, 1.18]	1.07 [0.97, 1.21]	0.07
FEF25-75	1.63 [1.23, 1.95]	1.54 [1.03, 1.88]	1.21 [0.98, 1.51]	1.48 [1.25, 1.70]	0.16
PEF	2.98 [2.58, 3.50]	3.20 [2.59, 3.65]	2.46 [2.16, 2.99]	2.66 [2.51, 3.17]	0.07
FEV1/FVC	0.85 [0.79, 0.88]	0.82 [0.77, 0.86]	0.82 [0.81, 0.88]	0.81 [0.77, 0.86]	0.43
FEV0.5/FVC	0.63 [0.58, 0.69]	0.60 [0.55, 0.65]	0.62 [0.56, 0.65]	0.57 [0.55, 0.65]	0.24
Total lgE (median [25^th^, 75^th^])	38 [19, 92]	49 [15, 103]	149 [20, 331]	218 [103, 475]	0.001
Any FEIA	40/91 (44%)	16/29 (55%)	11/15 (73%)	17/20 (85%)	0.003
Aero FEIA	38/91 (42%)	16/29 (55%)	11/15 (73%)	17/20 (85%)	0.002
Perennial aero FEIA	34/91 (37%)	15/29 (52%)	11/15 (73%)	16/20 (80%)	0.001
Seasonal aero FEIA	23/91 (25%)	7/29 (24%)	4/15 (27%)	9/20 (45%)	0.33

*FEV1* Forced Expiratory Volume at 1 second, *FEV1*%*pred* FEV1 percent predicted, *FVC* forced vital capacity, *FVC*%*pred*, FVC percent predicted; *FEV0.5*, Forced Expiratory Volume at 0.5 second, *FEF25-75*, Forced expiratory flow at 25% to 75% of FVC; *PEF*, peak expiratory flow rate; *Aero FEIA*, aeroallergen fluoroenzyme immunoassay.

### Peripheral blood Treg cell frequency is increased in children with asthma

**Figure 1:**
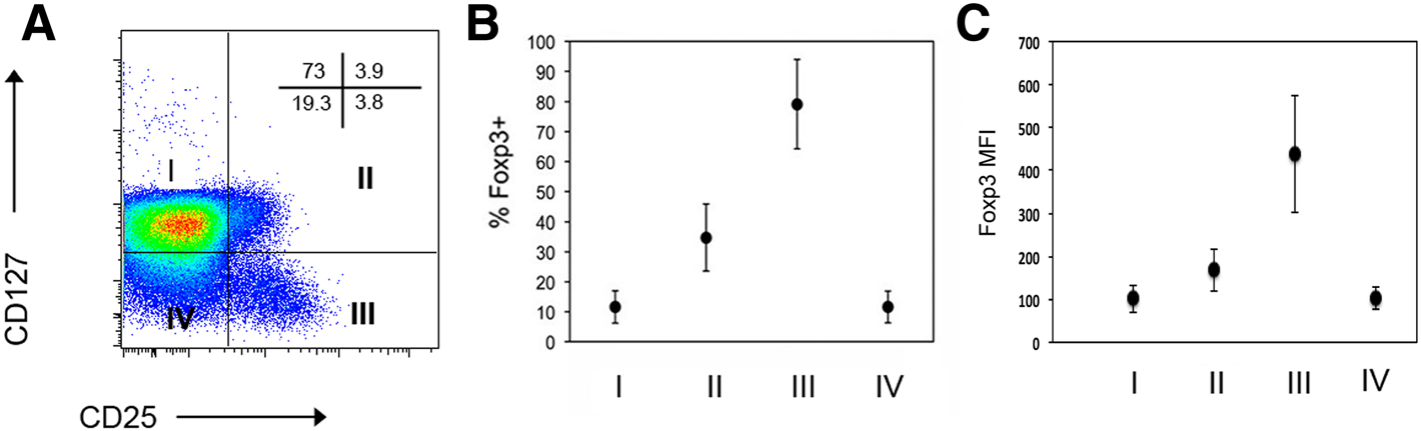
**CD4 + CD25 + CD127lo/- T cells express the majority of high level Foxp3 protein.** PBMCs were isolated and stained for flow cytometry as described in the methods section. Lymphocytes were gated using forward and side scatter profiles followed by CD3 and CD4 gating. **A**. Within this defined gate, CD127 and CD25 staining was determined using fluorescence minus one (FMO) controls. One representative subject is shown out of 159 assayed with the percentage of each population in upper right corner. **B**. Foxp3 staining was determined using an isotype control. The percentage of Foxp3 staining within each defined quadrant is plotted for the entire cohort (mean% of Foxp3+ cells within CD3 + CD4 + CD25 + CD127lo/-: 71.1%; SD 14.7%). The CD127 + CD25+ T cells have a subset of low expressing Foxp3+ cells (mean% of Foxp3+ within the CD3 + CD4 + CD25 + CD127+: 20.8%; SD 11.1%). **C**. Mean Fluorescent Intensity (MFI) for Foxp3 is shown. CD25 + CD127lo/- cells display the highest amount of Foxp3 per cell. The mean MFI for Foxp3 in CD3 + CD4 + CD25 + CD127lo/- T cells: 377 SD 137.8 vs. CD3 + CD4 + CD25 + CD127+ T cells: 146 SD 49.4.

We first sought to define the optimal approach for determining Treg cell frequency in a pediatric population. We analyzed PBMC by multi-parameter flow cytometry using a combination of phenotypic markers for Treg cells. In our cohort, similar to published work in adults [25], the CD4 + CD25 + CD127lo/- T cells expressed the majority of Foxp3 protein (Figure 1A & B). Foxp3 protein was detected in the CD4 + CD25 + CD127+ T cells (Figure 1B); however Foxp3 expression per cell was lower than in CD4 + CD25 + CD127lo/- T cells (Figure 1C). This finding is consistent with low level Foxp3 being an activation marker in effector T cells [26-31]. Based on these findings, we used the combination of CD25, CD127, and Foxp3 to define Treg cells.

**Figure 2:**
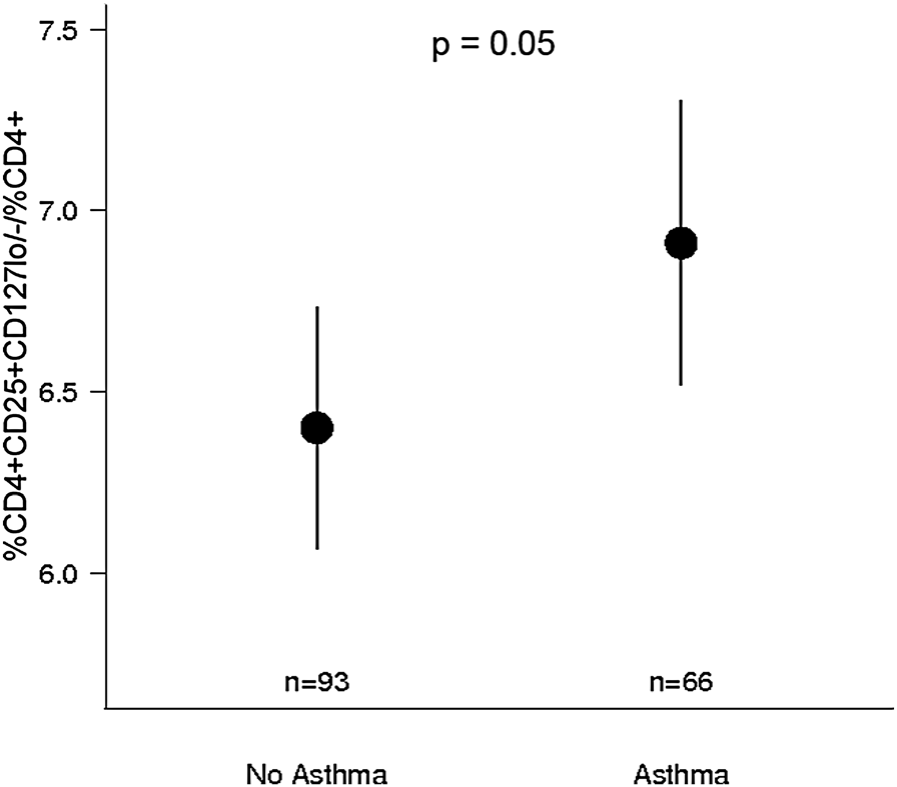
**Peripheral blood Treg cells are increased in children with asthma.** The percentage CD3 + CD4 + CD25 + CD127lo/- T cells in the peripheral blood of asthmatics (6.91% [6.52, 7.3]) versus control (6.4% [6.07, 6.73]) children. Data are expressed as the mean percentage of Treg cells within gated CD3 + CD4+ T cells with 95% CI.

Analysis of PBMC revealed a significantly increased percentage of CD4 + CD25 + CD127lo/- T cells in children with asthma compared to non-asthmatics (Figure 2). Interestingly, the Treg cell frequency was significantly higher in boys compared to girls (girls: 6.1 [5.7-6.5, 95%CI] vs. boys: 7.0 [6.7-7.3]; p = 0.001, data not shown). After adjustment for gender, increased Treg cell frequency remained significantly associated with a diagnosis of asthma. There was no significant difference in the percentage of Foxp3 positive cells within the CD4 + CD25 + CD127lo/- T cells or the mean fluorescent intensity (MFI) of Foxp3 between the groups (data not shown).

### Increased peripheral blood Treg cell frequency is associated with inhaled corticosteroid use and not asthma disease severity

**Figure 3:**
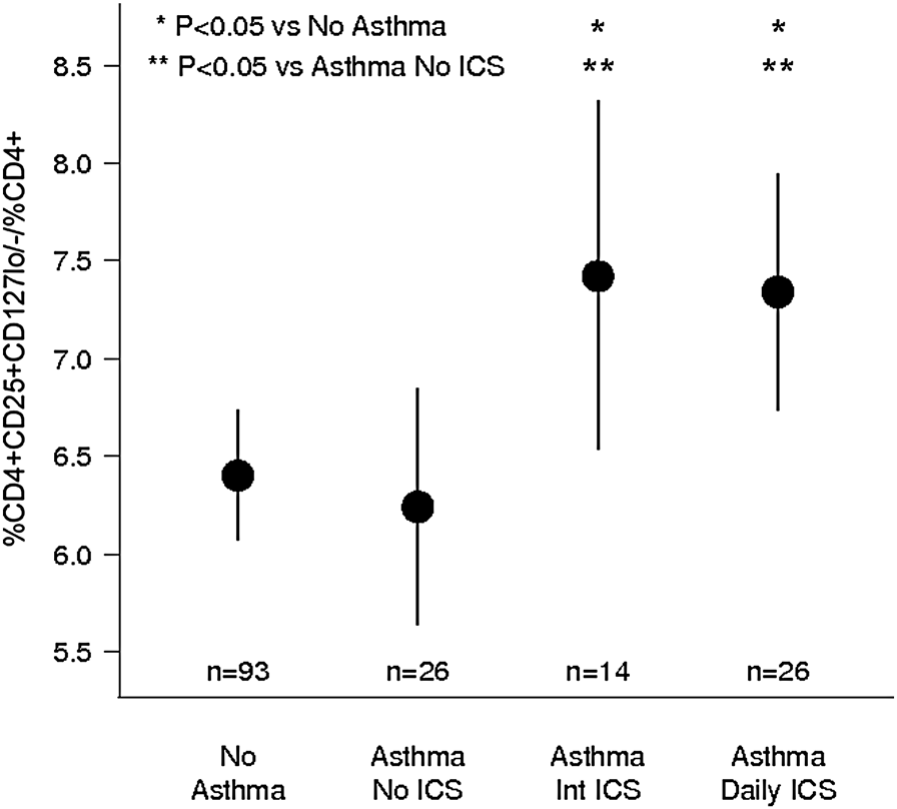
**Peripheral blood Treg cells are increased in asthmatic children using inhaled corticosteroids independent of asthma severity.** The percentage of Treg cells in the peripheral blood is elevated in asthmatics treated intermittently (Int) or daily with ICS. Data are expressed as the percentage of Treg cells within gated CD3 + CD4+ T cells with 95% CI. No asthma: 6.4% (6.08, 6.73); Asthma no ICS: 6.24% (5.64, 6.84); Asthma Int ICS: 7.42% (6.54, 8.31); Asthma daily ICS: 7.34% (6.74, 7.94).

We considered several possibilities for the observed increased frequency of Treg cells including a compensatory mechanism for airway inflammation or a secondary medication effect. Subjects were therefore categorized based on asthma disease severity and medication use. When asthmatic children were stratified based on inhaled corticosteroid (ICS) use, the increased frequency of Treg cells was positively related to intermittent or daily ICS use (Figure 3). When ICS use and asthma disease severity were considered in a 2-factor model, ICS use but not asthma severity was associated with increased Treg cell frequency (Table 2). ICS use remains significantly associated with increased Treg cell frequency after adjustment for gender (p = 0.001). Study subjects with documented respiratory viral infections tended to have higher Treg cells, though this was not significant (+0.7, 95% CI −0.5 to +1.9, p = 0.28) and ICS use is still significantly associated with higher Treg cell frequency after adjusting for infection.

**Table 2:** Increased Treg cell frequency is associated with ICS use and not asthma disease Severity

**Patient Classification**	**n**	**% Treg cells/CD4+ cells (mean, adjusted)**	effect*	**95%CI**	**p- value**
Children not on ICS	119	6.32	--	--	0.02
Children treated with ICS	40	7.37	1.06	(0.15, 1.96)	
No asthma	93	6.93	--	--	0.96
Intermittent	31	6.77	-0.17	(-0.91, 0.57)	
Mild persistent	15	6.77	-0.17	(-1.15, 0.82)	
Mod/Sev persistent	20	6.91	-0.02	(-1.17, 1.12)	
Interaction: P = 0.78					

*ICS* Inhaled corticosteroid, 95% CI, 95% confidence interval.

*differences in least squares (LS) means in%Treg cells/CD4+ cells between the groups.

### Peripheral blood Treg cell frequency is increased in children with allergen sensitization

Allergic sensitization is a major risk factor associated with the childhood asthma [32], but studies investigating the association between Treg cells and allergic sensitization have been conflicting [33-35]. To better understand the relationships among Treg cells, allergic sensitization and asthma, we examined the association between total IgE levels, allergen-specific sensitization, and Treg cell frequency. We found a positive correlation between serum total IgE levels and peripheral blood Treg cell frequency (R = 0.24, p = 0.003, data not shown). In addition, there was a trend for increased Treg cells in sensitized children (FEIA negative: 6.37% [5.99, 6.75] vs. FEIA positive: 6.82% [6.48, 7.17], p = 0.09, data not shown). When these two factors were considered in the same model, ICS use but not allergic sensitization was associated with an increased frequency of Treg cells (Table 3).

**Table 3:** Increased Treg cell frequency is associated with ICS use and not allergic sensitization

**Patient Classification**	**n**	**% Treg cells/CD4+ cells (mean, adjusted)**	effect*	**95%CI**	**p-value**
Children not on ICS	116	6.38	--	--	0.003
Children treated with ICS	39	7.32	0.94	(0.32, 1.55)	
FEIA negative	71	6.76	--	--	0.51
FEIA positive	84	6.94	0.18	(-0.35, 0.71)	
Interaction: P = 0.93					

ICS, inhaled corticosteroid; FEIA, fluroenzyme immunoassay; 95% CI, 95% confidence interval.

*difference in LS means in%Treg cells/CD4+ cells between the groups. Four children missing FEIA data were excluded from the analysis.

Lastly, we asked whether relationships between ICS and asthma depended on atopic status. When non-atopic asthmatics were separated from atopic asthmatics, the increased frequency in Treg cells was associated with ICS use, regardless of atopic status (Table 4). Subjects with asthma were more likely to have rhinitis (74% vs. 42%), data not shown, and asthma with ICS use had higher rates of rhinitis compared to asthma without ICS (83% vs. 62%). However, when adjusting the analysis for rhinitis, we still find that Treg cell frequency is significantly higher with ICS use (p = 0.005).

**Table 4:** Increased Treg Cell Frequency is Associated with ICS Use and Not Asthma Status

**Patient Classification**	**n**	**%Treg cells/CD4+ cells (mean, adjusted)**	effect*	**95%CI**	**p-value**
Children not on ICS	118	6.30	--	--	0.005
Children treated with ICS	39	7.55	1.26	(0.40, 2.12)	
No asthma	93	7.03	--	--	0.68
Non-atopic asthma	20	7.05	0.02	(-0.80, 0.84)	
Atopic asthma	44	6.69	-0.34	(-1.18, 0.50)	
Interaction: P = 0.35					

*ICS* Inhaled corticosteroid, 95% CI, 95% confidence interval.

*difference in LS means%Treg cells/CD4+ cells between the groups. Two asthma subjects are missing FEIA data and were excluded from the analysis.

## Discussion

In this study, we characterized the frequency of Treg cells in the peripheral blood of 159 children. To the best of our knowledge, this is the largest number of allergic asthmatic children analyzed using multi-parameter flow cytometry for Treg cell frequency determination. Our findings demonstrated a statistically significant association between increased frequency of Treg cells and intermittent or chronic ICS use. This association was independent of asthma disease severity, allergic sensitization, gender, BMI, or other atopic disease status. These findings indicate that ICS use is associated with an elevation in Treg cell frequency and that intermittent ICS use is sufficient for this systemic change to occur. This increase in Treg cell frequency observed with ICS use represents 1% of the total CD4+ cells and approximately 17% of the Treg cells. Moreover, these data suggest that inhaled corticosteroids may work by shifting the balance of adaptive immune responses toward Treg cell predominance. Lastly, our findings suggest that ICS treatment is an important covariate to consider in studies of Treg cells in asthma.

In this study, CD4 + CD25 + CD127lo/- T cells were used to define Treg cells. Recent work from our group utilizing multi-parameter flow cytometry alongside the demethylation status of a region of the Foxp3 promoter (Treg specific demethylated region,TSDR) found the strongest positive relationship using CD4 + CD25 + CD127lo/- T cells as the Treg cell defining phenotype when compared to CD4 + CD25 + CD127lo/-Foxp3+ T cells [36]. This finding suggests Foxp3 protein levels may not be constantly detectable using flow cytometry in bona fide Treg cells. Thus, multi-parameter flow cytometry using CD25 and CD127 to define Treg cells is reliable for quantification of CD4+ T cells expressing high levels of Foxp3 protein and demethylation at TSDR.

The impact of corticosteroids on Treg cells in asthma has not been extensively studied. It has been reported that corticosteroid exposure leads to increased Foxp3 mRNA expression in peripheral blood CD4+ T cells from adult asthmatics treated with oral and inhaled corticosteroids [37]. Smyth et al. demonstrated an increased frequency of CD4 + Foxp3+ T cells in the bronchial alveolar lavage fluid (BALF) in adult patients with moderate-to-severe asthma compared to mild asthmatics and healthy controls, but did not account for medication use or atopic status of the patients [18]. In our study, all of the moderate-to-severe asthmatics were on ICS, and a subset of the study subjects (16%) had one or more bursts of oral corticosteroids (OCS) during the study period. In contrast to ICS, the use of OCS was not significantly associated with increased Treg cell numbers (p = 0.80, data not shown). Our findings in peripheral blood suggest that the increased frequency of BALF Treg cells in the study by Smyth et al. might also be secondary to ICS use. Hartl et al. studied a small number of children with symptomatic asthma (n = 18) demonstrating diminished BALF Treg cell, defined as CD4 + CD25hi, frequency with normalization after initiation of ICS use compared to control groups [19]. Yuksek et al. noted an increase in peripheral blood Treg cells, defined as CD4 + CD25hiFoxp3+, in a small number of asthmatic children (n = 16) after initiation of inhaled corticosteroids [38]. In general, our data are in agreement with the observation that ICS use increases Treg cell frequency, and indicate that there are similar changes in the blood and airway. Our data further clarifies the relationship of ICS and Treg cells using a more rigorous definition of Treg cells with multi-parameter flow cytometry, an increased sample size, and presents a more thorough characterization of other atopic characteristics (allergic sensitization, asthma severity, atopic status).

Several mechanisms could contribute to a positive association between ICS use and increased Treg cells. For example, corticosteroids enhance Foxp3 transcription [37,39]. Alternatively, ICS could alter homing characteristics of Treg cells, or represent a compensatory response to dampen ongoing lung inflammation. Similarly, the positive association between serum IgE and Treg cell frequency could represent a compensatory response to allergic inflammation. Additional studies are warranted to investigate longitudinal changes in Treg cells before and after ICS use. These lines of investigation would help support a mechanism of action for ICS and may serve as a prognostic indicator of response to therapy.

Recently, several studies have implicated Treg cell dysfunction in allergic asthmatics [4,19]. Further analysis of Treg cells in our cohort, including quantifying the percentage of Foxp3+ T cells within the defined Treg cell population and MFI of Foxp3, did not reveal any significant differences with these indirect measurements of Treg cell function. This remained consistent when subjects were segregated by asthma, disease severity, or ICS use (data not shown). Studies further phenotyping the Treg cell compartment and analyzing functional characteristics are ongoing in our laboratory.

## Conclusions

In summary, our study demonstrates an increased frequency of Treg cells in the peripheral blood of asthmatic children using ICS. This relationship suggests that corticosteroid use should be considered when analyzing the frequency of Treg cells in asthma. The modest increase (~17%) in Treg cell frequency was observed even with intermittent ICS use. These findings suggest the possibility that ICS-mediated increases in Treg cell frequency could contribute to the beneficial ICS effects on asthma disease control.

## Abbreviations

PBMC: Peripheral blood mononuclear cells; Treg: T regulatory cell; FEIA: Fluoroenzyme immunoassay; ICS: Inhaled corticosteroids; COAST: Childhood Origins of ASThma.

## Competing interests

No competing of interests exist for any of the authors.

## Authors’ contributions

All authors contributed to data acquisition for the study, AMS, RFL, JEG and CMS contributed to the design, analysis of the study, and manuscript preparation. MDE and RG did all statistical analyses. CMS is the guarantor of this article. All authors read and approved the final manuscript.

## Supplementary Material

Additional file 1 Asthma classification of subjects and medication use. Click here for file
