# Supplementary material for: Inhaled corticosteroid use is associated with increased circulating T regulatory cells in children with asthma
Source: Clin Mol Allergy. 2013 Jan 25;11:1. doi: 10.1186/1476-7961-11-1 (PMC3598778; doi:10.1186/1476-7961-11-1)
Supplement: Additional file 1 — Asthma classification of subjects and medication use. [file 1476-7961-11-1-S1.docx]

**Table 2: Asthma Classification of Subjects and Medication Use**

| **Subject** | **Lowest Daily Medication Required for Asthma Control** | **Asthma Classification** | **Intermittent ICS*** |
| --- | --- | --- | --- |
| 17 | Leukotriene receptor antagonist | Mild persistent |  |
| 24 | No daily medication | Intermittent |  |
| 26 | ICS plus long-acting beta agonist | Moderate persistent |  |
| 31 | no daily medication | Intermittent |  |
| 36 | Leukotriene receptor antagonist | Mild persistent |  |
| 40 | Leukotriene receptor antagonist | Mild persistent |  |
| 48 | Medium dose ICS | Moderate persistent |  |
| 50 | No daily medication | Intermittent |  |
| 52 | No daily medication | Intermittent |  |
| 55 | No daily medication | Intermittent |  |
| 58 | No daily medication | Intermittent |  |
| 67 | No daily medication | Intermittent |  |
| 68 | No daily medication | Intermittent |  |
| 70 | No daily medication | Intermittent |  |
| 75 | No daily medication | Intermittent |  |
| 79 | ICS plus leukotriene receptor antagonist | Moderate persistent |  |
| 99 | Low dose ICS | Mild persistent |  |
| 100 | No daily medication | Intermittent | Yes |
| 111 | Low dose ICS | Mild persistent |  |
| 116 | No daily medication | Intermittent |  |
| 118 | Medium dose ICS | Mild persistent |  |
| 121 | Medium dose ICS | Moderate persistent |  |
| 124 | No daily medication | Intermittent |  |
| 125 | No daily medication | Intermittent |  |
| 146 | No daily medication | Intermittent | Yes |
| 147 | No daily medication | Intermittent | Yes |
| 154 | No daily medication | Intermittent | Yes |
| 162 | No daily medication | Intermittent |  |
| 178 | No daily medication | Intermittent | Yes |
| 184 | No daily medication | Intermittent | Yes |
| 185 | No daily medication | Intermittent | Yes |
| 186 | No daily medication | Intermittent | Yes |
| 199 | No daily medication | Intermittent |  |
| 200 | Leukotriene receptor antagonist | Mild persistent |  |
| 205 | Medium dose ICS | Moderate persistent |  |
| 212 | No daily medication | Intermittent |  |
| 217 | Leukotriene receptor antagonist | Mild persistent |  |
| 218 | Leukotriene receptor antagonist | Mild persistent |  |
| 225 | No daily medication | Intermittent | Yes |
| 252 | Leukotriene receptor antagonist | Mild persistent | Yes |
| 263 | No daily medication | Intermittent | Yes |
| 264 | Low dose ICS | Mild persistent |  |
| 271 | No daily medication | Intermittent |  |
| 281 | No daily medication | Intermittent | Yes |
| 296 | No daily medication | Intermittent | Yes |
| 298 | No daily medication | Intermittent | Yes |
| 409 | ICS plus long-acting beta agonist | Severe Persistent |  |
| 410 | Medium dose ICS plus leukotriene receptor antagonist | Moderate persistent |  |
| 422 | ICS plus long-acting beta agonist | Moderate persistent |  |
| 423 | Medium dose ICS plus leukotriene receptor antagonist | Moderate persistent |  |
| 439 | Low dose ICS | Mild persistent |  |
| 442 | ICS plus leukotriene receptor antagonist | Moderate persistent |  |
| 444 | Low dose ICS | Mild persistent |  |
| 445 | Low dose ICS | Mild persistent |  |
| 446 | ICS plus long-acting beta agonist plus leukotriene receptor antagonist | Severe Persistent |  |
| 448 | Medium dose ICS plus leukotriene receptor antagonist | Severe Persistent |  |
| 449 | ICS plus leukotriene receptor antagonist | Moderate persistent |  |
| 450 | ICS plus long-acting beta agonist plus leukotriene receptor antagonist | Severe Persistent |  |
| 451 | Medium dose ICS plus leukotriene receptor antagonist | Moderate persistent |  |
| 453 | Leukotriene receptor antagonist | Mild persistent |  |
| 454 | ICS plus leukotriene receptor antagonist | Moderate persistent |  |
| 455 | ICS plus long-acting beta agonist | Moderate persistent |  |
| 456 | ICS plus leukotriene receptor antagonist | Moderate persistent |  |
|  |  |  |  |
| **Patients Not Adequately Controlled**** | |  |  |
| **Subject** | **Current level of medication** | **Classification** | **Impairment** |
| 283 | No daily medication | Moderate persistent | nightly nocturnal symptoms, >2x/week daytime |
| 452 | Low dose ICS | Moderate persistent | >2 nights/month |

*Children on no daily medication, but who used ICS for step-up therapy only where considered intermittent ICS users. ** Children for whom medication use alone did not indicate severity at the time of visit. Symptoms are summarized in the table.
